# Oncoplastic and reconstructive breast surgery

**DOI:** 10.3389/fonc.2023.1176915

**Published:** 2023-06-28

**Authors:** Primeera Wignarajah, Charles M. Malata, John R. Benson

**Affiliations:** ^1^Department of Breast Surgery, Royal Marsden Hospital NHS Trust, London, United Kingdom; ^2^Department of Breast Surgery, Cambridge Breast Unit, Hospital, Cambridge University Hospitals NHS Foundation Trust, Cambridge, United Kingdom; ^3^Department of Plastic and Reconstructive Surgery, Hospital, Cambridge University Hospitals NHS Foundation Trust, Cambridge, United Kingdom; ^4^Anglia Ruskin University School of Medicine, Cambridge/Chelmsford, United Kingdom

**Keywords:** breast reconstruction, oncoplastic breast surgery, breast implants, fat grafting, autologous free flap, nipple sparing mastectomy

## Abstract

This article provides an overview of the principles and techniques of oncoplastic and reconstructive breast surgery for patients with early-stage breast cancer. Oncoplastic breast surgery (OPBS) with partial breast reconstruction is a natural evolution in the application of breast conserving surgery and permits wide surgical resection of tumours that might otherwise mandate mastectomy and whole breast reconstruction. These reconstructive techniques must be optimally selected and integrated with ablative breast surgery together with non-surgical treatments such as radiotherapy and chemotherapy that may be variably sequenced with each other. A multidisciplinary approach with shared decision-making is essential to ensure optimal clinical and patient-reported outcomes that address oncological, aesthetic, functional and psychosocial domains. Future practice of OPBS must incorporate routine audit and comprehensive evaluation of outcomes.

## Part 1

## Introduction – Breast cancer epidemiology

Breast cancer remains the most common malignancy worldwide with recent lifetime estimates of 1 in 7 in the United Kingdom (UK) (1) where the annual number of cases has almost doubled in the past four decades. Globally, one-quarter of female cancers have breast as the primary site and the World Health Organisation (WHO) reported in 2020 that 2.3 million women worldwide were diagnosed with breast cancer and more than 600,000 women died from their disease (2). Within the UK, almost half of breast cancers are diagnosed within the screening age bracket and introduction of the screening programme led to a surge in incidence that was confined to women of initial screening age (3). Breast cancer is a disease predominantly of post-menopausal women and rising rates during the final decade of the last century has been attributed to increased usage of hormone replacement therapy (HRT) amongst affluent women (4, 5). Use of exogenous hormones in women aged 45-69 years fell dramatically after 2002 and resulted in a transient reduction in breast cancer incidence amongst white American women but this decline has not been sustained despite limited contemporary usage of HRT (6, 7). Genetic factors are likely to be more important for breast cancer development in younger women with increasing recognition of lower penetrance genes that individually confer lower levels of risk but are collectively important. Breast cancers frequently display epigenetic phenomena that permit changes in gene expression without DNA sequence alterations and thereby acts as translators between the external environment and the genome.

Those countries that historically had moderate or low rates of breast cancer based on income levels are now experiencing rapid rate increases with an inexorable rise in the incidence of breast cancer in China and India and a doubling of rates in Japan over the past 50 years. The high incidence rates in Western industrial nations have been attributed to lifestyle factors that now have relevance to increasing rates amongst emerging economies. These include changes in reproductive behaviour, altered dietary habits with increased consumption of polyunsaturated fats and alcohol together with a more sedentary lifestyle and physical inactivity (8–10). These are potentially modifiable risk factors and breast cancer incidence could be significantly reduced by adoption of a healthier lifestyle with maintenance of optimum body weight, limited alcohol intake and regular exercise (11–13).

Mortality rates for breast cancer have fallen over the past 30 years despite a continued rise in incidence. This testifies to the success of interventional strategies such as screening and adjuvant systemic therapies that permit diagnosis of breast cancer prior to formation of micrometastatic disease or obliteration of established foci of disease at distant sites. Survival rates at 10 years for breast cancer in the UK are currently 80.4% compared with approximately 55% in the final quarter of the last century (1). Survivorship has become an important issue with an estimated 7.8 million patients around the world living with breast cancer diagnosed in the past 5 years (14). Survival rates will continue to improve with advances in translational research and development of tailored therapies (e.g antibody drug conjugates) that can effectively target micrometastatic disease with acceptable levels of serious side-effects – there is a balance between length and quality of life. Many women with breast cancer typically diagnosed when in their fifties are now surviving well into their eighties and any adverse effects of treatment (surgery, radiotherapy, chemotherapy, oestrogen deprivation) can have a lasting impact on the remaining period of the patient’s life. With improved clinical outcomes for breast cancer treatments including both disease-free and overall survival, the focus has now shifted to quality-of-life issues. Although many studies have confirmed that aesthetic results of breast cancer surgery are a principal determinant of quality-of-life and patient satisfaction, functional and psychosocial outcomes are equally important and should be part of any shared decision-making process (15, 16).

## Development of oncoplastic breast surgery

William Stewart Halsted (1852-1922) published the first formal description of an operation for breast cancer based on a series of patients treated at the Johns Hopkins Hospital in Baltimore, USA (17). He postulated that breast cancer is a loco-regional disease and metastatic dissemination occurs by centrifugal and contiguous spread of the primary tumour with progressive involvement of adjacent tissue and the lymphatic system of the breast. The operation of radical mastectomy aimed to remove *en bloc* the breast, pectoral musculature, and the axillary lymph nodes (up to level III). This operation was rapidly implemented as routine surgical practice for breast cancer patients in the first half of the twentieth century irrespective of clinical features (assuming the tumour was operable). A fundamental concept of this so-called *Halstedian paradigm* was that maximal efforts at local control would prolong survival; breast cancer was considered to originate as a localised disease, and it was surmised that cure rates could be improved by a more meticulous and comprehensive surgical approach. Local recurrence was considered to be the cause of distant metastases and the aim was to minimise rates of local relapse. Halsted observed that many patients developed local recurrence before they succumbed from distant metastatic disease. His operation of radical mastectomy reduced rates of local recurrence from 60% to 6% but had no impact on overall survival – so this mutilating operation did not provide patients with any additional years of life. There was a problem with the existing paradigm; hence an alternative hypothesis was proposed by the eminent surgeon Bernard Fisher (1918-2019) whose brother Edward (‘Ed”) was a pathologist. This paradigm was known as biological pre-determinism and contended that breast cancer is a local manifestation of a systemic disease with complex interactions between the host, the primary tumour and distant micrometastases (18). Breast cancer was considered capable of accessing the circulation at an early stage in carcinogenesis with cancer cells breaking away from the tumour bolus and entering the bloodstream *via* holes between the endothelial cells in the neovasculature. In addition, haematogenous dissemination was possible *via* lymphatico-venous communications in the regional (axillary) lymph nodes. It followed that surgery could only achieve local control of disease and some form of systemic treatment was necessary to improve overall survival (19). This paradigm of Fisher was supported by results of six randomised prospective trials that allocated breast cancer patients to either a breast conservation procedure (lumpectomy, wide local excision, quadrantectomy) or total mastectomy. The first of these trials was conducted by Umberto Veronesi (1925-2016) at the National Cancer Institute of Milan, Italy (20) and the largest trial (NSABP B-06) (21) by Bernard Fisher under the auspices of the National Surgical Adjuvant Breast and Bowel Project in Pittsburgh. Results of the Milan I trial appeared on the front cover of the New York Times in 1981 and provided level I evidence demonstrating survival equivalence for breast conservation therapy compared with radical or modified radical mastectomy (22). An update of the NSABP B-06 trial with a 20 year follow up confirmed that post-operative irradiation improved local recurrence-free survival after breast conserving surgery (BCS) with similar distant disease-free and overall survival for modified radical mastectomy, wide local excision and radiotherapy or wide local excision alone (21). Hence permutations of breast surgery had no impact on breast cancer-specific mortality and BCS was deemed to be a safe surgical procedure for patients with tumours <5cm in size. This heralded the start of a trend for de-escalation of breast surgical procedures. Residual cancer cells are a determinant of local failure but not of distant metastatic disease with a finite rate of ipsilateral breast tumour recurrence (IBTR) when BCS is undertaken. Contemporary rates of IBTR are very low (<1% per annum) with combined multimodality treatments; systemic therapies reduce IBTR by approximately one-third and anti-HER2 directed treatments halve rates of in-breast recurrence. BCS represents a balance between oncological mandates and cosmetic outcomes with the aim of removing the tumour and a narrow margin of surrounding breast tissue such that negative margins are achieved. There is now international consensus that an adequate margin exists when tumour is not touching ink and wider margins do not reduce rates of local recurrence (23). Nonetheless, the Association of Breast Surgery (UK) have decreed that a negative margin requires tumour to be no closer than 1mm from the inked margin for both invasive and non-invasive breast cancer. A negative margin does not imply absence of any residual disease within the remaining breast tissue but indicates a residual tumour burden sufficiently low to be controlled with adjuvant treatments. Local surgery does not completely eliminate residual disease with local recurrence determined by a combination of surgery, tumour biology, radiation and systemic therapies (24).

A spectrum or intermediate paradigm is emerging which encompasses this variable capacity to form distant metastatic disease, with more indolent, slower growing tumours (luminal subtypes) behaving according to the *Halstedian paradigm* and more aggressive tumours (triple negative and HER2 positive cancers) disseminating early on – consistent with the *Fisherian paradigm* (25). Molecular profiling of tumours has revealed a dichotomy of gene expression patterns that permits assignment of tumours to one or other group based on predicted biological behaviour with appropriate intensities of loco-regional and systemic treatments.

The modified radical mastectomy removed breast and axillary tissue in continuity but preserved the pectoralis major muscle and much reduced the morbidity of the traditional radical operation. This operation was championed by David Patey of the Middlesex Hospital in London but was never widely adopted outside the UK (26). With the rapid development of breast reconstructive techniques over the past three decades, the modified radical mastectomy has evolved into skin-sparing and nipple-sparing forms of mastectomy that are now being applied to both prophylactic and therapeutic breast surgical procedures. Skin-sparing mastectomy (SSM) was introduced by Toth and Lappert in 1991 (27); initial concerns that greater skin preservation might lead to higher rates of local recurrence have not been justified. Several studies have now confirmed low rates of local recurrence (<5%) for skin-sparing procedures are not significantly higher than for conventional forms of mastectomy when patients are matched for stage of disease (28, 29). Nipple-sparing mastectomy (NSM) is the ultimate form of conservative mastectomy in which the entire breast envelope is preserved. Ongoing studies are attempting to define those breast cancer patients for whom NSM can be safely performed for the ipsilateral breast without adversely affecting oncological outcomes, especially recurrence within the territory of the nipple. It is particularly important that ductal tissue within the nipple is ‘cored’ out without compromising the vascular supply to the nipple-areola complex (30).

Axillary surgery is an integral component of breast cancer surgery and has undergone a revolutionary change with progressive de-escalation of nodal resection. The operations of both radical and modified radical mastectomy implied concomitant axillary lymph node dissection (ALND). However, with the advent of sentinel lymph node (SLN) biopsy, formal ALND is now much less commonly performed (as either a primary or a secondary procedure); a notable change has been omission of completion ALND in selected sentinel node positive cases with reliance on adjuvant non-surgical treatment modalities for eradication of low burden axillary disease in non-sentinel lymph nodes (31). The majority of patients nowadays undergo initial SLN biopsy, be this in the context of conventional mastectomy without reconstruction, SSM, NSM or BCS. Thus patients are more likely to undergo simple mastectomy combined with SLN biopsy rather than mastectomy and ALND – the modified radical mastectomy.

The development of oncoplastic surgery and partial breast reconstruction is a natural evolution in the application of BCS to management of breast cancer. Most patients who are considered eligible for BCS have a favourable tumour to breast size ratio and are suitable for conventional forms of wide local excision with local glandular readjustment but no formal remodelling of the breast. Even when re-excision of margins is required (in up to one-quarter of cases), an optimal cosmetic outcome should be attainable in the long term after irradiation of the breast. There is a ‘grey area’ where the limits of BCS are being approached and the patient may be better served with a skin/nipple-sparing mastectomy and immediate breast reconstruction at the outset. It becomes progressively more difficult to achieve a good cosmetic outcome as the proportion of breast tissue removed increases. When more than 10%-20% of breast tissue is removed, there is a risk of an unsatisfactory result, but relatively modest losses of 5%-10% of breast volume from tumours in cosmetically sensitive areas (medial and inferior quadrants) can adversely affect cosmesis (32). Oncoplastic breast surgery (OPBS) provides the opportunity for enhancing quality-of-life by improving cosmetic outcomes and psychological wellbeing after larger resections for unifocal and some multifocal breast cancers. OPBS can facilitate wide surgical clearance of a tumour and improve a patient’s cosmetic outcome when larger volumes of resected tissue are required (33). Techniques for OPBS include volume replacement and volume displacement techniques (34). The former imports additional tissue in the form of a flap and attempts to compensate for loss of volume from surgical excision. By contrast, the latter rearranges the remaining breast tissue using methods of glandular advancement or rotation that serve to redistribute the parenchyma and minimise the impact of wide local excision. Volume displacement techniques absorb volume loss over a wider area and do not incur donor site morbidity from harvesting of any local tissue flaps. Werner Audretsch from Dusseldorf in Germany is credited with pioneering many of these techniques for OPBS and incorporating techniques of aesthetic plastic surgery into routine breast surgery for partial breast reconstruction after extirpative procedures for breast cancer. Audretsch coined the term ‘oncoplastic surgery’ and worked closely with colleagues such as Krishna Clough in Paris and Richard (Dick) Rainsbury in UK to establish OPBS techniques and define indications for use of a variety of different techniques depending on the size of the tumour, location within the breast and whether uni- or multifocal (35, 36). The advent of oncoplastic techniques has very much defined the ethos of current approaches to breast surgery and acceptance that a good cosmetic result should be standard of care for all patients without compromise of oncological safety. A particular challenge is the integration of adjuvant treatments into management pathways for breast cancer patients with determination of optimum sequencing and timing of chemotherapy and radiotherapy. These techniques remain contentious, and Clough has referred to the oncoplastic ‘frenzy’ (37); careful selection of patients is crucial and partial breast reconstruction should not be attempted in patients who are not amenable to BCS from an oncological perspective and for whom mastectomy is warranted (38). These techniques of OPBS must be appropriately integrated with ablative breast surgery to avoid emergence of a ‘breast cripple’. Cross-specialty training opportunities are fostering increasing numbers of oncoplastic breast surgeons and those without oncoplastic competencies should work co-operatively with plastic surgeons to provide a comprehensive service. Notwithstanding availability of surgical expertise, these OPBS techniques are relevant to a relatively small proportion of patients (10-15%) although indications for the use of oncoplastic techniques are increasing and breast surgeons are accruing more experience with these techniques for the benefit of patients (39). All ‘breast surgeons’ are in a sense ‘oncoplastic’ and whatever their precise breed must work co-operatively with plastic surgeons to ensure that a mix of surgical skills can be optimally applied to maximise oncological, aesthetic and patient reported outcomes.

The ‘coming of age’ of OPBS (40) has allowed many women to benefit from management planning by a multidisciplinary team offering a comprehensive breast cancer and reconstructive service. By restoring the size, shape and appearance of the breast, reconstruction improves a patient’s gender identity and quality-of-life across multiple domains – psychological, social, sexual, emotional, and functional. The breast is a symbol of femininity and its characteristic curves have defined the female form throughout the ages. In addition, there is enhanced aesthetic satisfaction compared with mastectomy alone (41), and some patients with borderline conservable tumours may opt for mastectomy and whole breast reconstruction rather than BCS with partial breast reconstruction. Some women steadfastly want to keep their breast if at all possible, whilst others are adamant they want a mastectomy (often with a contralateral prophylactic mastectomy-so called ‘big surgery’) despite having a small tumour. It is therefore imperative that patients make fully informed decisions and are aware of reconstructive options early on in discussions of surgical management of their breast cancer. Breast surgical oncologists have a key role as gatekeepers in ensuring that patients have access to reconstructive surgeons and referrals to plastic surgery colleagues are made as soon as possible following diagnosis of breast cancer – including patients managed with primary chemotherapy. Interestingly, there is evidence that patients are significantly more likely to be referred for reconstruction if breast surgeons are female, have a high workload and are affliated with a designated cancer centre (42). Decisions made in terms of breast reconstruction are very much ‘preference sensitive’ and must be individualised and made jointly between a patient and her surgeon(s). They should take account not only of a patient’s wishes, but also her personality, self-perception, hobbies, family, and socio-economic circumstances. There may be a selection bias with OPBS patients tending to be younger with higher levels of educational attainment and income that may influence their self-perception and attitudes towards body image. Outcomes of breast cancer surgery are better when clinical decision making integrates information giving, shared decision-making and patients’ personal values. There is now greater appreciation of a patient’s perspective and issues such as quality-of-life and patient choice. These complement traditional outcomes based on objective surgical criteria and are being formally measured with validated questionnaire-based instruments such as BREAST-Q that can measure more subjective outcomes related to psychological, emotional, and functional sequelae of reconstruction (43). Incorporation of patient reported outcome measures (PROMS) with more objective clinical parameters will inform future patient choice and lead to improvements in clinical care. Clinical decision making in the field of surgical oncology and OPBS has become increasingly complex in recent years and involves multidisciplinary team working and integration of a large number of variables requiring collective assessment before planning surgery. As previously mentioned, many treatment options are based on low levels of evidence that is of poor quality and often outdated and not necessarily related to contemporary practice. Artificial intelligence offers the potential opportunity to more accurately assess this complex array of variables and aid the clinical-decision making process; this can avoid personal judgement and bias and the adage ‘my way and the wrong way’. Methodologies such as GRADE and Delphi interviews attempt to reconcile potentially conflicting viewpoints and assimilate opinion and experience from a large number of clinicians and place this in context with published data. The technique of text–mining can be employed to analyse decision drivers (44). Surgeons and other healthcare workers must be honest with patients when discussing cosmetic and other outcomes of breast reconstructive surgery. In particular, patients’ expectations must be realistic and appreciate that the reconstructed breast is a facsimile of a normal breast. It is important to stress to the patient it is a ‘breast mound’ that is being created rather than a ‘breast’. Whenever possible, patients should be offered a full repertoire of reconstructive options but the final surgical procedure undertaken will depend on several factors including surgeon experience and training, general health of the patient (including co-morbidities and smoking habits) and local healthcare resources.

A patient’s expectations of the final cosmetic result will be determined by any relevant prior knowledge and influenced by information derived from family, friends, other patients, the media and increasingly the internet. Any misperceptions must be corrected, and fully informed consent obtained before proceeding with any form of breast reconstruction. Levels of patient satisfaction are often more related to adequate information and a robust shared decision-making process than an aesthetically pleasing cosmetic result.

There must be alignment between the aims of the surgeon and the patient – the latter takes comfort and reassurance from a surgeon’s knowledge and skills. Establishment of a good rapport will encourage shared decision-making and lead to optimal outcomes. Patients must be given sufficient time to assimilate all information and reach a decision that feels right for them; sometimes they will change their mind, and this must be accommodated. Surgeon preference should not dominate discussions and patients should be offered a full repertoire of reconstructive options and not compelled to accept a reconstructive option that is especially favoured by her surgeon (for whatever reason). Surveys have revealed that patients who chose reconstruction are motivated by body image rather than reasons relating to sexuality or femininity (45). By contrast the most common reason for patients declining breast reconstruction is to avoid additional surgery (45). Higher levels of patient satisfaction are associated with immediate breast reconstruction compared with mastectomy alone in terms of psychosocial, sexual and physical well-being (46, 47). Nonetheless, a desire to complete adjuvant cancer treatments prior to reconstructive surgery is a frequently cited reason for patients deliberately opting for a delayed breast reconstruction.

Increasingly breast units around the world are employing specialist breast care nurses as well as dedicated breast reconstruction nurses. These individuals exercise a valuable role in clarifying and processing information for patients. They can sometimes help frame relevant questions ahead of any consultation with the surgeon and this will facilitate shared decision-making. Introduction of separate oncoplastic multidisciplinary team meetings combines the expertise of breast surgeons, plastic surgeons, radiologists, and medical/radiation oncologists. It is important that these potentially problematic OPBS cases are discussed jointly between breast surgeons, plastic surgeons and oncologists to determine optimal management. Treatments are increasingly tailored to individual patients and based on tumor phenotype. Most triple negative and HER2 positive tumors >2cm will be managed with primary chemotherapy and in the latter case anti-HER2 therapy. Complete pathological response rates often exceed 50% and concentric shrinkage of tumors will facilitate subsequent surgery with breast conservation being an option instead of mastectomy (with or without whole breast reconstruction). Individual cases can be discussed in depth with access to clinical notes, radiological images and medical photography. Oncoplastic multidisciplinary team meetings are being widely adopted and used to aid in surgical decision making and providing options for patients. These meetings are also an excellent forum for trainees.

The range of OPBS options in the modern era is considerable and the potential choice of options available to a woman with a newly diagnosed breast cancer can be overwhelming. Extreme oncoplastic breast conserving surgery (EOBCS) refers to the use of oncoplastic breast conservation techniques in patients with multifocal, multicentric or locally advanced breast cancer (>5cm) which would be conventionally treated with mastectomy (+/- reconstruction). EOBCS has evolved with the improvements in systemic therapies, radiotherapy techniques and increased awareness of the psychological and quality-of-life benefits of breast conservation. In 2015 Silverstein et al. reported 66 cases of EOBCS in patients who were advised mastectomy and declined. There is a lack of long-term data on the impact on recurrence, overall survival and distant metastases in patients opting for EOBCS (48, 49). There is arguably no longer a primary decision of BCS versus mastectomy with or without reconstruction. Instead, two key questions are whether the patient is a candidate for conventional BCS and if not, can she be spared mastectomy with either pre-operative chemotherapy or an oncoplastic procedure? (50) Addressing these questions may involve complex surgeon-patient discussions with viewing of radiological images (preferably correlation with MRI) and anonymised before and after photographs of previous patients. These discussions will involve patient tailoring measurements and conceptualised diagrams. The possibility of retaining or improving shape whilst replacing or displacing breast tissue with OPBS may be an option. Alternatively, there will always be the option of removing all breast tissue with preservation of much of the skin envelope and whole breast reconstruction with prosthetic material, autologous tissue, or a combination thereof. The relative advantages and disadvantages of each option must be discussed with patients including surgeon-specific complication rates. It should be remembered that OPBS is not plastic surgery per se, but the use of plastic surgical techniques and principles to improve outcomes of cancer treatment. Patients should appreciate that surgery is only one aspect of their management pathway and other treatment modalities will affect the final cosmetic results.

Historically there has been a dearth of high quality research in OPBS with minimal level I evidence derived from randomised controlled trials – the latter are arguably more challenging to undertake in this field. This often relates to issues of patient and surgeon preference in terms of specific operative procedures that can undermine surgical equipoise and dissuade patients from accepting treatment options determined by a process of randomisation. A more pragmatic trial design for evaluation of clinical and patient reported outcomes is prospective observational studies whereby patients can chose a particular surgical option and different groups of patients will then be compared in terms of specific outcome measures. This type of design is subject to confounding from unmeasured bias but otherwise represents a way of encouraging trial participation and relatively rigorous evaluation of outcomes whilst allowing patients to chose their surgery (irrespective of how this might be influenced by their surgeon’s personal procedural preference). Failure of trials to randomise OPBS patients provides valuable insights into how future clinical trials in this field should be designed (51).

## Genetics, breast cancer and reconstruction

There has been a flurry of public interest in genetic testing and risk reduction strategies following revelations that the actress Angelina Jolie had chosen to undergo bilateral prophylactic mastectomy due to carriage of a BRCA-1 gene mutation. In addition to increased demand for genetic testing amongst breast cancer patients, the number of contralateral prophylactic mastectomy (CPM) cases have increased three-fold since this story appeared in the New York Times in 2013 (52). Genetic counselling and testing for breast cancer predisposition has been formally implemented in many countries and the number of women seeking genetic testing continues to rise. In the UK, The National Institute for Health and Clinical Excellence (NICE) has recommended that women undergo genetic testing when the chance of finding a high-penetrance mutation is 10% (reduced from the previous mandate of 20%) (53). Most women over-estimate their risk and genetic testing allows accurate risk assessment that more confidently informs any proposed management decisions. Nonetheless, despite these advances in genetics, approximately 30% of familial breast cancer risk remains unaccounted for by mutations in currently known genes. Moreover, genetic changes do not necessarily have a causative association with a diseased state and phenotypic manifestations of cancer are variable. The clinical management of women with an asymptomatic mutation in BRCA1 and BRCA2 or carriage of a mutation in other high-risk genes such as TP53, PALB2 or pTEN is increasingly complex but often those with a pathogenic mutation in a high-risk gene will seek bilateral risk-reducing mastectomy (RRM) (with or without immediate breast reconstruction).The PALB2 gene encodes for a protein that interacts with the BRCA-2 gene product to repair damaged DNA and maintain fidelity of DNA replication. Mutations of the PALB2 gene are associated with a breast cancer risk of 35 - 40% by age 70 years that is slightly lower than for BRCA-2 mutations where the comparable risk is 40 – 60% (54). Increasing numbers of patients with PALB2 mutations are being referred from clinical genetics for consideration of bilateral RRM. Most patients with mutations in high/moderate risk genes are relatively young and seek immediate breast reconstruction – hence genetic testing has led to increased demand for reconstructive breast surgery. However, the risks associated with bilateral RRM must be carefully balanced against benefits in terms of reduced incidence (not mortality) of subsequent breast cancer (>90%) and psychological advantages with alleviation of uncertainty and concomitant anxiety. All patients undergoing prophylactic surgery (including CPM) must receive appropriate counselling and have a formal psychological assessment.

Genetic testing in the UK can take up to 12 weeks before results become available and management of younger women with primary chemotherapy (triple negative/HER2 positive breast cancer) provides a convenient surgical pause allowing genetic test results to be available when planning definitive surgical treatment. Some younger women without a documented pathogenic variant but a strong family history of breast/ovarian cancer may opt for risk-reducing surgery.

## Part 2

The second part of this article will address the different types of techniques available for OPBS and criteria for selection of patients for appropriate options. The latter must ensure optimal oncological, and patient reported outcomes whilst minimising complications and delays in commencement of adjuvant treatments.

## What are the aims of oncoplastic surgery in management of breast cancer patients?

The challenge of oncoplastic surgery is to reconcile oncological and aesthetic outcomes and maximise levels of patient satisfaction. There are broadly three primary aims that need to be addressed for each patient:

1) Optimal oncological outcome – performing an extirpative procedure that minimises the chance of recurrence by removing the tumour with a clear margin of normal surrounding breast tissue as part of either a partial or complete mastectomy.
2) Optimal cosmetic outcome – reconstituting the breast with either partial or whole breast reconstruction to provide optimal symmetry and shape in relation to the native breast.
3) Minimal delays in commencement of adjuvant treatments – prevention of post-operative complications such as infection, wound dehiscence, haematoma, seroma, or fat necrosis that interfere with delivery of adjuvant treatment such as radiotherapy and chemotherapy.

Reconstructive breast cancer surgery includes both mastectomy and whole breast reconstruction as well as partial breast reconstruction employing a variety of oncoplastic techniques that have been developed by breast surgeons and often drawn from plastic surgery principles. Oncoplastic breast surgery (OPBS) encompasses volume rearrangement, volume displacement and volume replacement techniques. *Volume rearrangement* involves the use of local tissues to optimize the shape after wide local excision. It incorporates careful incision planning, appropriate undermining of the breast skin, meticulous closure of the dead space and mobilization of the local tissues. *Volume replacement* imports additional tissue with a flap to compensate for loss of volume from surgical ablation. By contrast, *volume displacement* rearranges the remaining breast tissue using methods of glandular advancement/rotation/transposition that serve to redistribute parenchyma and minimize the cosmetic impact of tumour excision. This is also referred to as ‘therapeutic mammoplasty’. In effect, the volume loss is absorbed over a wider area with concomitant re-shaping of the breast. Volume displacement surgery is less complex than autologous tissue transfer methods and avoids associated donor site morbidity. The reconstructed breast is notably of smaller volume and plastic surgery on the contralateral side is often required for symmetrization, which (more often than not) is an integral part of therapeutic mammoplasty. This applies especially to therapeutic mammoplasty where tumour excision is incorporated into standard/modified reduction procedure or breast lift. Volume displacement represents the simplest option for partial breast reconstruction and is usually preferred over techniques for volume replacement that involve more extensive surgery with harvesting of a myocutaneous or subcutaneous perforator flap. Volume displacement techniques are preferably used in patients with medium to large breasts with a significant degree of ptosis that render these patients well suited to these techniques. By contrast, volume replacement techniques are indicated in small breasted women (48, 49, 55).

## Partial breast reconstruction

Techniques for OPBS were formally classified by Krishna Clough in 2010 (35) and divide procedures into two categories based on the extent of breast tissue resection and degree of surgical complexity for reconstruction of the conserved breast:

*Level 1 OPBS techniques* - these involve resection of at least 20% of total breast volume that require relatively straightforward volume displacement techniques to achieve an acceptable cosmetic result with reshaping of the breast through advancement, rotation or transposition of existing parenchyma and skin with a resultant decrease in overall breast volume.
*Level 2 OPBS techniques* – these involve resection of between 20% and 50% of total breast volume with restoration using methods for either displacement or replacement of breast tissue that may be combined with skin reduction or transfer.

### Level 1 OPBS

When up to 20% of breast volume is resected without any attempt to mobilize and re-model adjacent glandular tissue, then a significant defect in breast contour, shape and size may ensue. Resection of breast tissue in the upper outer or lower outer quadrants is less likely to result in a noticeable defect compared with resections in more cosmetically sensitive areas such as the upper inner quadrant. The cosmetic outcome after removal of a relatively small volume of tissue can be enhanced by simple mobilization of breast tissue adjacent to the surgical cavity. The extent of mobilization required will depend on the size of the defect and may involve undermining the whole breast plate. Extensive mobilization of breast tissue can sometimes threaten the blood supply to both the glandular tissue and skin. This can lead to post-operative necrosis and secondary infection with a poor aesthetic outcome and impaired quality-of-life (56). Therefore, mobilization and displacement of glandular tissue using advancement or rotational flaps to fill a defect presents an opportunity for improved cosmesis but can be technically challenging (57).

### Level 2 OPBS

*Volume displacement techniques* – these can be employed to adjust for loss of larger breast volumes (20 – 50%) and usually involve some form of mammoplasty that includes a variety of techniques such as Wise pattern, batwing, Grisotti, Benelli, Round block and vertical mammoplasty (LeJour) pattern.

**Figure 1:**
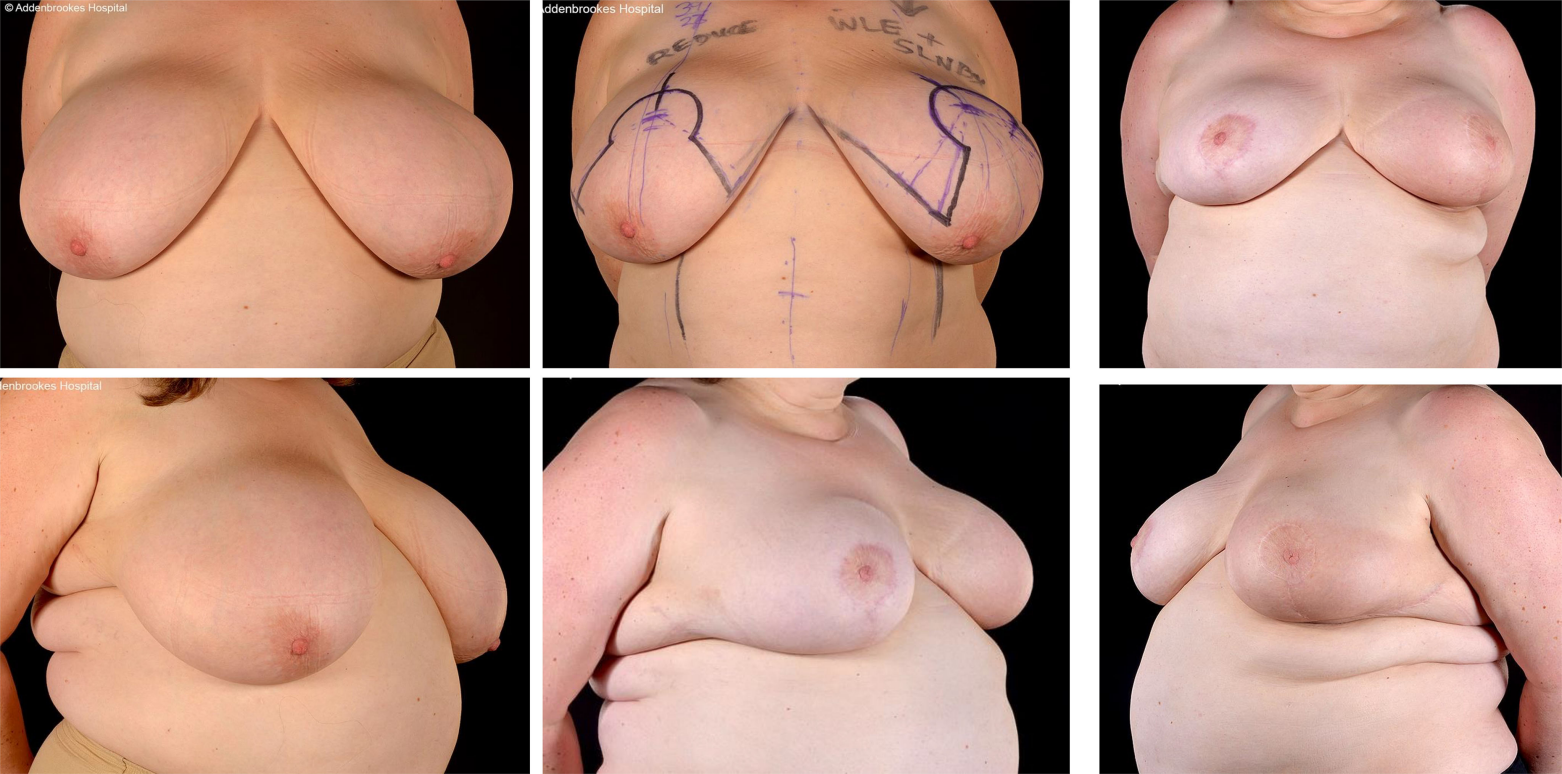
Left therapeutic mammaplasty in a patient with gigantomastia necessitating contralateral balancing breast reduction. Symmetrisation surgery is an integral part of therapeutic mammaplasty specifically and oncoplastic surgery in general.

**Figure 2:**
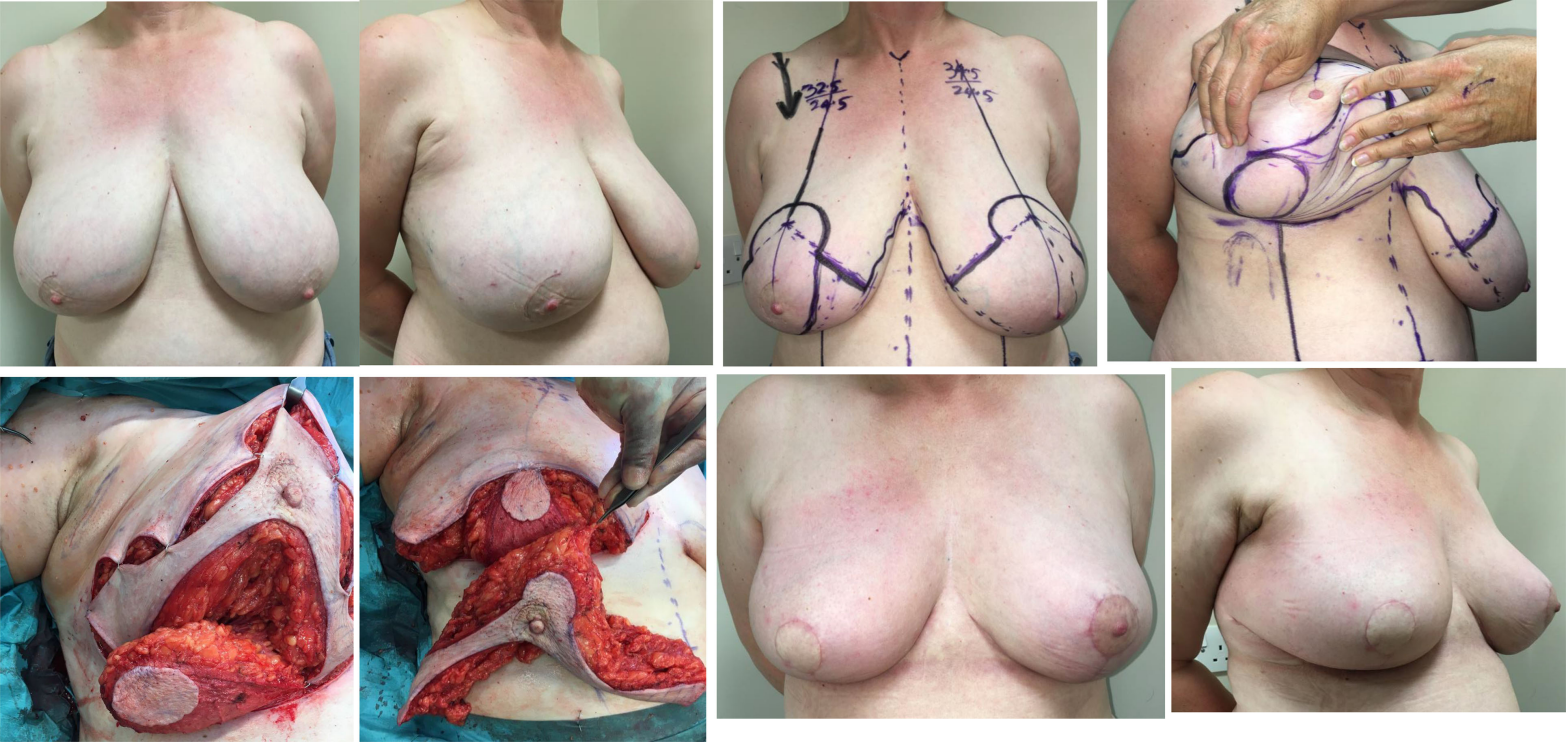
Right modified Grisotti flap based on the inferior pedicle, used for partial reconstruction to create a neo-areola after right therapeutic wise pattern mammoplasty with axillary clearance and left inferior pedicle based contralateral balancing reduction mammoplasty. Right therapeutic reduction weight 778g. Left breast reduction weight 842g.

**Figure 3:**
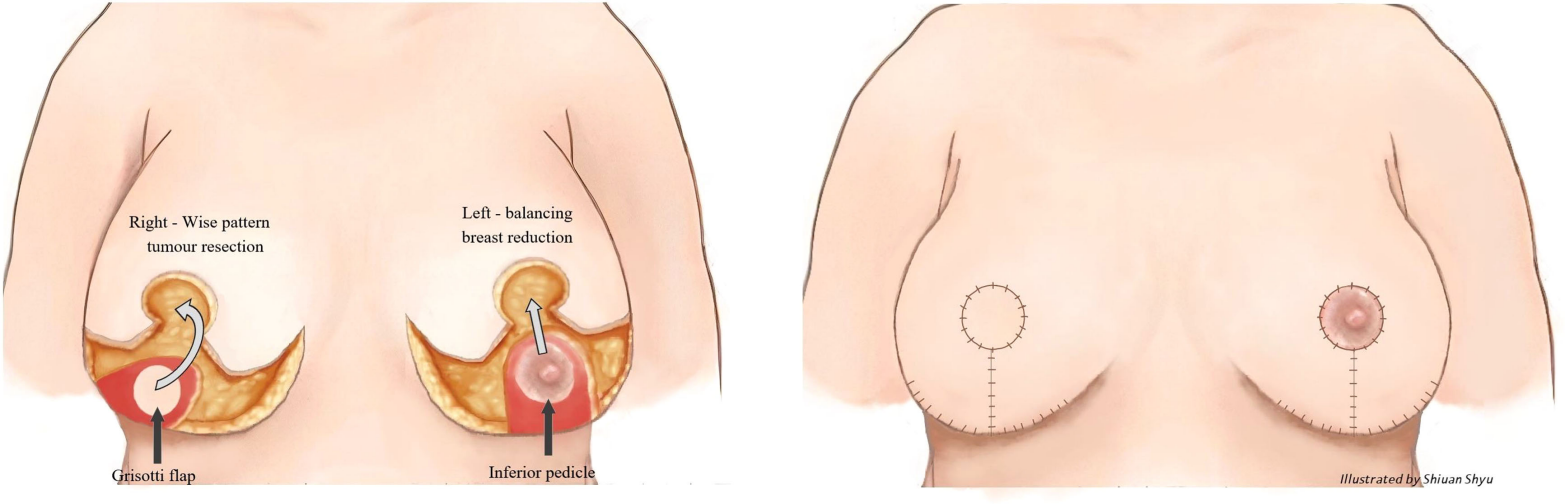
Right therapeutic wise pattern mammoplasty and contralateral symmetrising left breast wise pattern reduction mammoplasty using inferior pedicles. White arrows indicate the direction of flap rotation and final placement. Illustrated by Shiuan Shyu.

**Figure 4:**
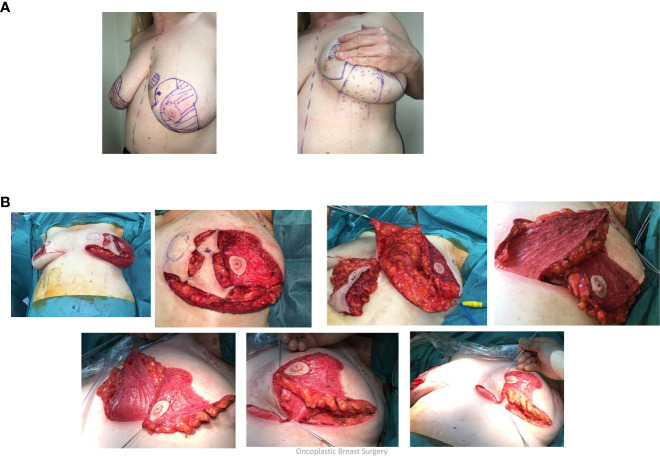
**(A)** Superolateral pedicle and secondary (de-epithelialised totally buried) inferior pedicle: Bilateral breast cancers – different locations, multifocal left breast cancer. **(B)** Intraoperative sequence.

These various mammoplasty techniques involve resecting the tumour and a pre-determined volume of tissue and skin with rearrangement of the glandular tissue to re-form the breast. The re-fashioned breast is often smaller and less ptotic than the native breast and contralateral breast surgery is frequently indicated for symmetrization (especially for high percentage excision of breast volume) – **Figure 1**. Depending on tumour location and disease extent, it may be feasible to preserve the nipple-areola complex on a defined pedicle but otherwise this structure may need to be sacrificed (in which case a partial breast reconstruction can be performed using a Grisotti flap) – **Figure 2**. An inferior pedicle technique (**Figure 3**) preserves a pyramid of tissue in the inferior portion of the breast to maintain perfusion of the nipple. This can potentially compromise the oncological resection volume for a tumour in the inferior portion of the breast and therefore a superior pedicle is more appropriate. The superior pedicles can be superolateral or superomedial (**Figure 4**). The batwing and hemi-batwing mammoplasty are used for tumours in the superior breast that are relatively close to the nipple-areola complex and involve less mobilization of glandular tissue yet permit excision of tumours with an adequate margin and a good cosmetic result. A symmetrizing procedure on the contralateral breast can be undertaken simultaneously with the therapeutic procedure or at a later date following completion of adjuvant treatments such as chemotherapy and radiotherapy – and allowing time for radiotherapy changes to settle.

**Figure 5:**
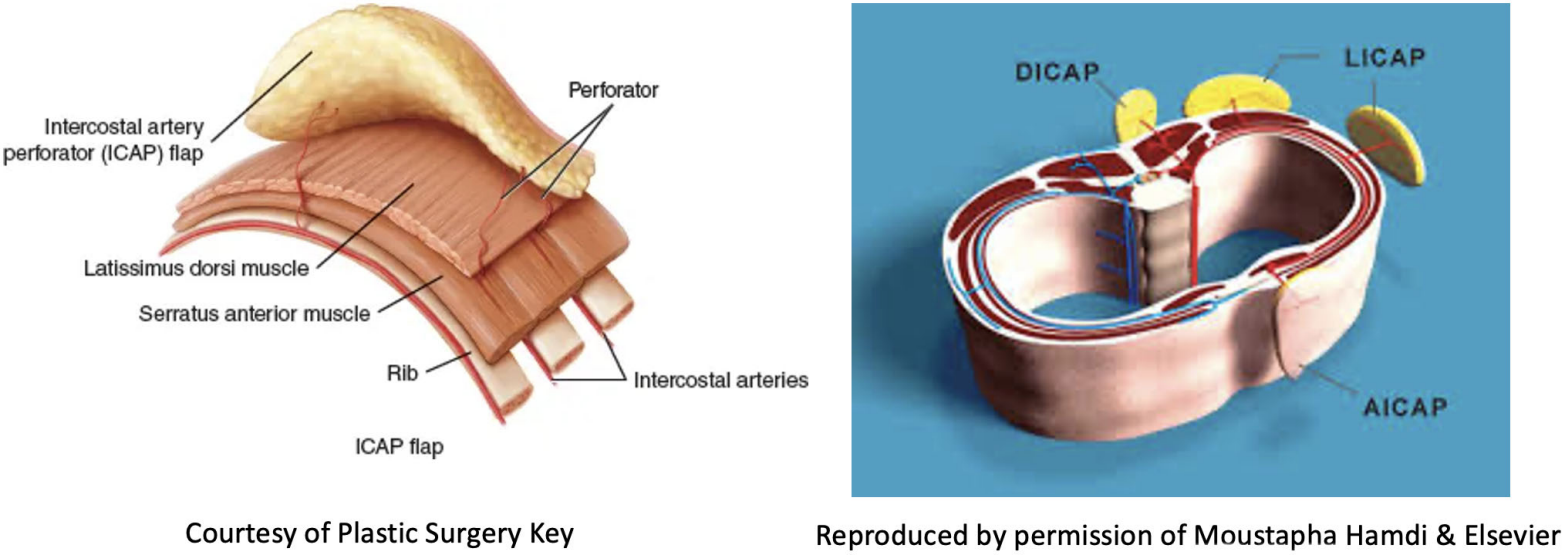
Schematic diagrams showing the intercostal artery perforator flaps: nomenclature and anatomy.

**Figure 6:**
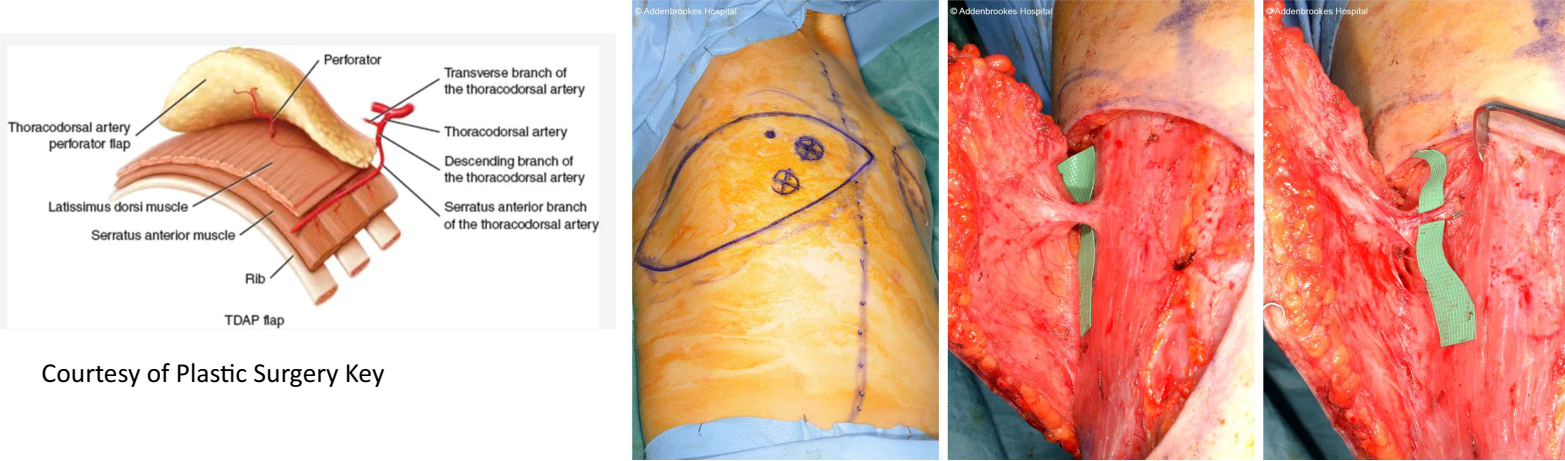
TDAP flap vascular anatomy and intraoperative harvest. Courtesy of Plastic Surgery Key. TDAP flap with key perforator 3cm posterior to the anterolateral border of the LD muscle. Intraoperative images show location of the perforator and the muscle split required to increase vascular pedicle length and increase its arc of rotation. No muscle is sacrificed. A small part of muscle can be sacrificed (type 1) vs type 2.

*Volume replacement techniques* – these have previously been reliant on use of the latissimus dorsi (LD) flap harvested as either a myocutaneous flap in the form of a standard LD flap or the mini-LD muscle flap, the latter popularized by Rainsbury (58). More recently replacement techniques have been increasingly based on local flaps such as chest wall perforator flaps. Chest wall perforator flaps have become popular in recent years as a method for partial breast reconstruction when larger volumes of tissue are resected in the inferior and lateral aspects of the breast (**Figure 5**). These highly specialized volume replacement techniques include the lateral intercostal artery perforator flap (LICAP), anterior intercostal artery perforator flap (AICAP), medial intercostal artery perforator flap (MICAP), lateral thoracic artery perforator (LTAP) flap as well as the thoracodorsal artery perforator which utilizes the same pedicle as the latissimus dorsi musculocutaneous or muscle flap (**Figure 6**) (59–62).

Although older forms of loco-regional flaps are associated with worse cosmetic results, these can be used on selected patients and permit breast conserving surgery to be undertaken based on random flaps (thoraco-epigastric, thoracolateral and bi-pedicled flaps).

**Figure 7:**
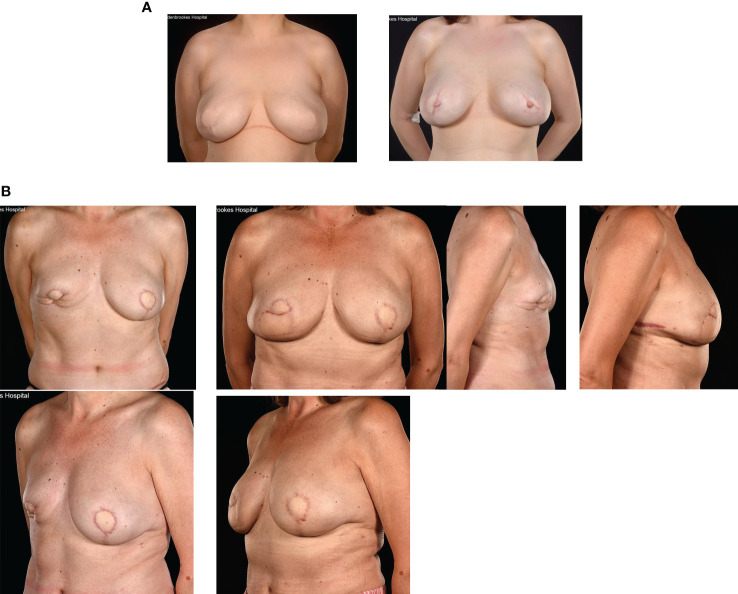
**(A)** Fat grafting of contour defects of bilateral LD + expandable implants for risk reducing surgery. **(B)** Fat necrosis of an immediate SIEA flap: treated by a TDAP flap and serial fat grafting from the abdomen and flanks.

Other methods for enhancing breast volume include lipo-modelling that has recently had a renaissance in the context of breast cancer surgery. This is especially useful as an adjunctive technique to ‘plump up’ mastectomy skin flap thickness around implants (**Figure 7A**) and restoring local volume defects following breast conservation (**Figure 7B**). Lipo-modelling (or fat grafting) involves harvesting fat from a donor site (lateral thigh, hip, lower back, buttocks or abdomen), filtering the fat and injecting it into the breast to improve volume, shape and symmetry. It can also be used to fill localized mastectomy flap defects after whole breast reconstruction. This is especially so after pre-pectoral (epi-pectoral) breast reconstruction when it is used to soften the sharp take off and pad the tissues around the implant. The benefits of fat grafting relate to its being bio-compatible and readily available coupled with its versatility and ability to integrate into host tissues and survive. Lipo-modelling can result in improved aesthetic outcomes but has some notable disadvantages, especially in terms of the donor site where there may be a defect, loose skin or cellulite-like appearance in the long term (63, 64). Moreover, short-term bruising and swelling can be associated with significant morbidity for patients. Only 40- 60% of the injected fat is likely to be retained at the recipient site with significant local absorption of grafted fat tissue (65, 66). There are also risks of fat necrosis and oil cyst formation and a requirement for multiple courses of lipo-modelling which often require general anaesthesia. Nonetheless, the advent of lipo-modelling has permitted correction of intractable aesthetic deformities and minor asymmetries that were previously difficult to ameliorate surgically. A notable usage is the improvement of the skin quality in radiotherapy-damaged skin either with lumpectomy or total breast reconstruction.

A wide range of OPBS techniques are now available and selection of the optimal method for any individual patient is dependent on breast size, degree of ptosis, tumour location (quadrant), the surgeon’s expertise and patient preference. In terms of patient satisfaction, it is important that expectations are realistic, and patients understand that their overall breast size many be smaller following oncoplastic breast surgery and scarring can be more extensive than anticipated for some level II OPBS techniques. The choice of OPBS technique will also be influenced by the surgeon’s skills set.

**Figure 8:**
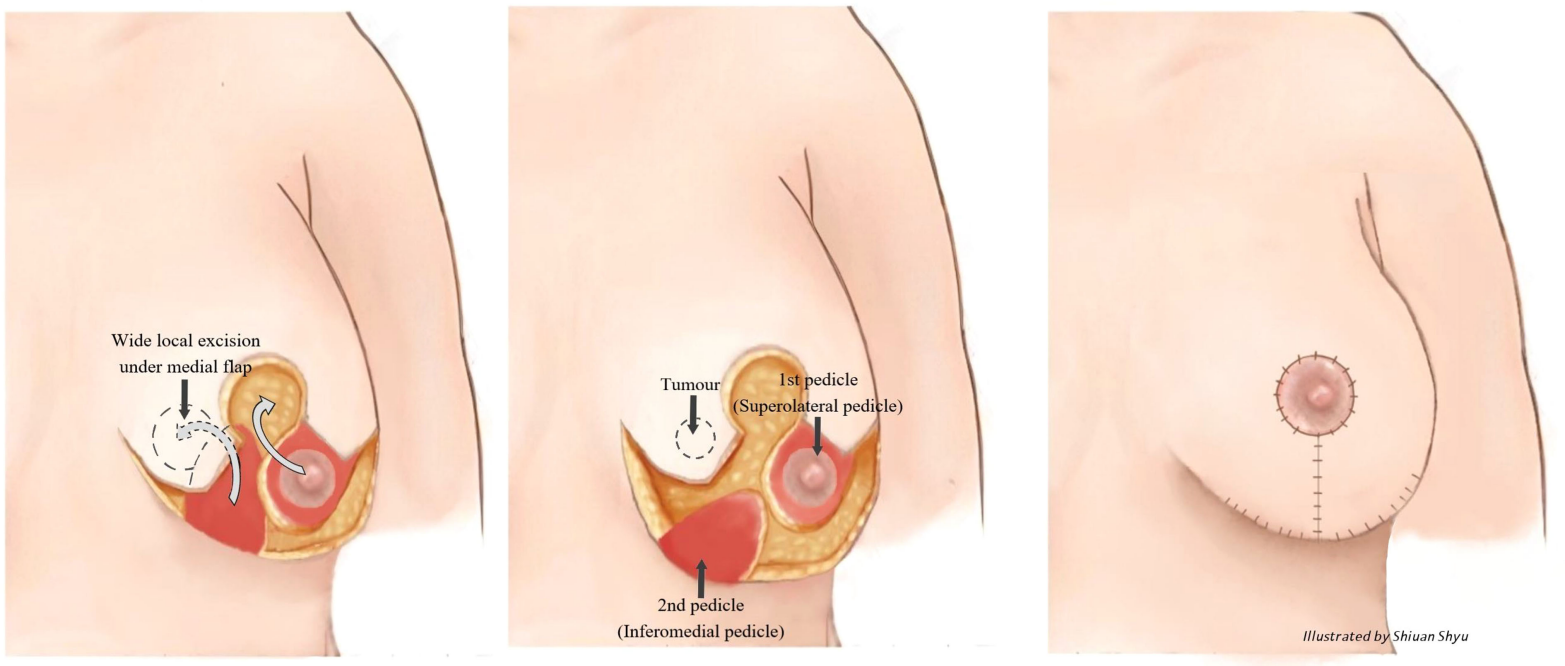
Superolateral pedicle and secondary (de-epithelialised totally buried) inferior pedicle. White arrows indicate the direction of flap rotation and final placement. Illustrated by Shiuan Shyu.

Careful planning of skin incisions and appropriate orientation of the nipple-areola complex pedicle is essential when performing more complex volume displacement techniques that demand detailed knowledge of the blood supply of the breast and appreciation of plastic surgery principles. This applies particularly to transposition of glandular tissue when there has been extensive undermining from both chest wall and skin and secondary pedicles have been created during a classical Wise-pattern (therapeutic) mammoplasty to address complex/extensive defects and avoid a mastectomy (**Figure 8**). The nipple-areola complex must be preserved on a robust pedicle – be this superior or inferior or variations of these. These more complex cases of OPBS often demand a multidisciplinary approach involving collaboration between plastic and breast surgeons for optimal outcomes.

**Figure 9:**
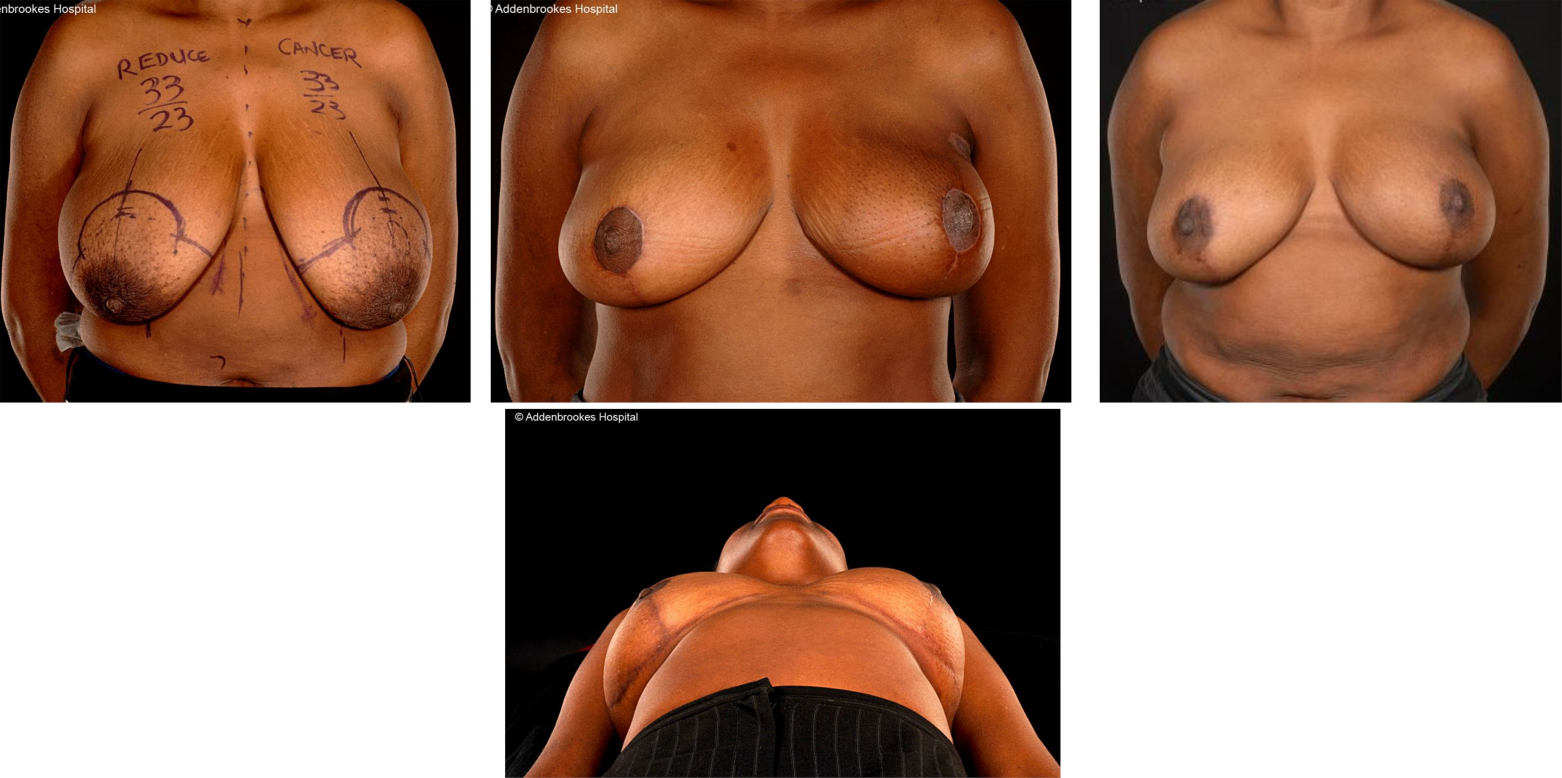
Effects of radiotherapy following inferior pedicle technique therapeutic mammaplasty in a 44 year old showing severe radiotherapy reaction and follow up 5 years later showing changes have largely settled.

It is important to take account of treatment effects upon surgical outcomes for OPBS; any reduction in final breast volume and shape can be unpredictable following radiotherapy (**Figure 9**). The ultimate aesthetic result may fall short of patient and surgeon expectations even in the absence of any technical challenges during surgery or complications thereafter.

## Whole breast reconstruction

In many parts of the world, reconstructive surgery is the exclusive remit of plastic surgeons, with breast surgeons undertaking the extirpative component of surgery only (namely skin/nipple-sparing mastectomy with axillary surgery). In the UK, breast surgeons are routinely trained in techniques of whole breast reconstruction that are implant-based with or without adjuvant material (matrix/mesh). NICE guidelines state that patients should be offered reconstruction unless existing co-morbidities pose a contraindication (67).

Breast reconstruction can be performed at the time of mastectomy (immediate breast reconstruction [IBR]) or at any time after mastectomy (delayed breast reconstruction [DBR]). In recent years, IBR has gained wider acceptance with improved cosmetic results and reassuring evidence that reconstruction does not mask detection of recurrent disease (68–72).

Furthermore, there are documented psychological benefits from IBR (73–76) and patients awake from anaesthesia with two breasts, albeit one being a facsimile of a breast (i.e a breast mound). By contrast, patients must endure being flat-chested on one or both sides for a period of time whilst awaiting DBR. Nowadays, patients most frequently request IBR and studies with PROMs have shown high levels of satisfaction for IBR (although some studies report higher scores for DBR as patients may compare the reconstructed breast with a flat chest rather than a native breast (77–79)).

For some patients, mastectomy is mandated for breast cancer treatment based on factors such as the tumour: breast ratio, multifocal/centric cancers, or perhaps failed attempts at breast conserving surgery. A decision for mastectomy may also relate to age, genetic predisposition, and availability of IBR. Not all patients seek IBR and this may be contraindicated for some patients based on cancer type (inflammatory cancers), co-morbidities, BMI and smoking history. When proceeding with IBR, one of the most important early decisions is whether the nipple-areola complex should be preserved, and this will be determined on grounds of oncological safety, aesthetic benefit, surgical feasibility, and patient wishes. If pre-operative imaging reveals no direct involvement of the nipple by tumour and there is sufficient distance to the nipple, then nipple preservation is usually feasible, and several studies have confirmed the safety of NSM in terms of local recurrence (80, 81). Nonetheless, some surgeons prefer to take nipple biopsies either pre-operatively with a needle or intra-operatively with frozen section examination of the specimen or intraoperative cores behind the nipple for paraffin sections.

Many breast units have strict selection criteria for IBR with smoking status and BMI being of paramount importance. A BMI in excess of 30 is associated with a 4-fold increase in major complications following IBR (82–84) but most breast units set a BMI threshold between 32 and 35 for IBR. It is essential that patients understand the relative risks and benefits associated with IBR, especially for autologous tissue reconstruction and show compliance with post-operative guidance/instruction.

**Figure 10:**
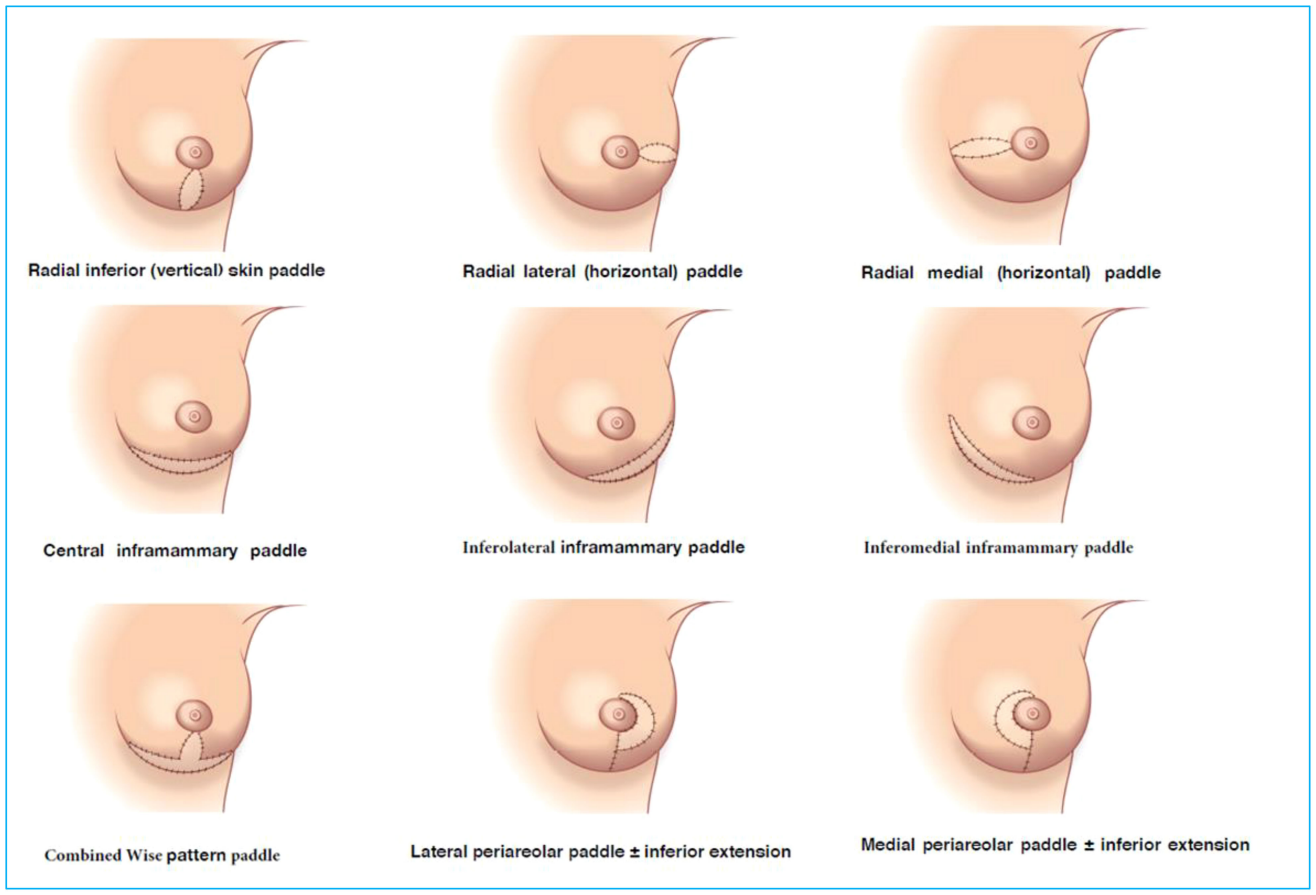
Access incisions for nipple-sparing mastectomies and flap reconstruction (85).

**Figure 11:**
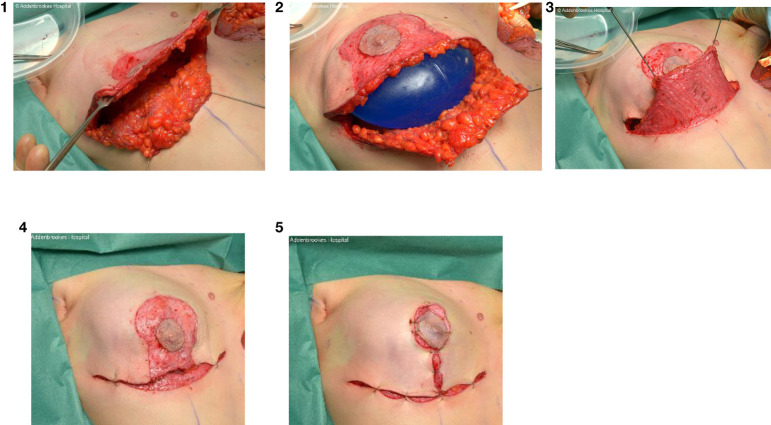
Intraoperative sequence of skin-reducing mastectomy with nipple-preservation, pre-pectoral implant and dermal sling reconstruction.

Skin incisions for skin- and nipple sparing mastectomy should be discussed between surgeon and patient as these are associated with different rates of complications but a peri-areolar incision (+/- medial/lateral extensions) and infra-mammary fold (IMF) incision respectively are conventionally used and preferred by the majority of surgeons (**Figure 10**). The IMF incision is generally used for smaller breasts with no pre-operative ptosis, but a radial incision can be employed for more central access to the breast parenchyma. For larger breasts it may be necessary to use special incisions for so-called skin-reducing mastectomies (**Figure 11**).

**Figure 12:**
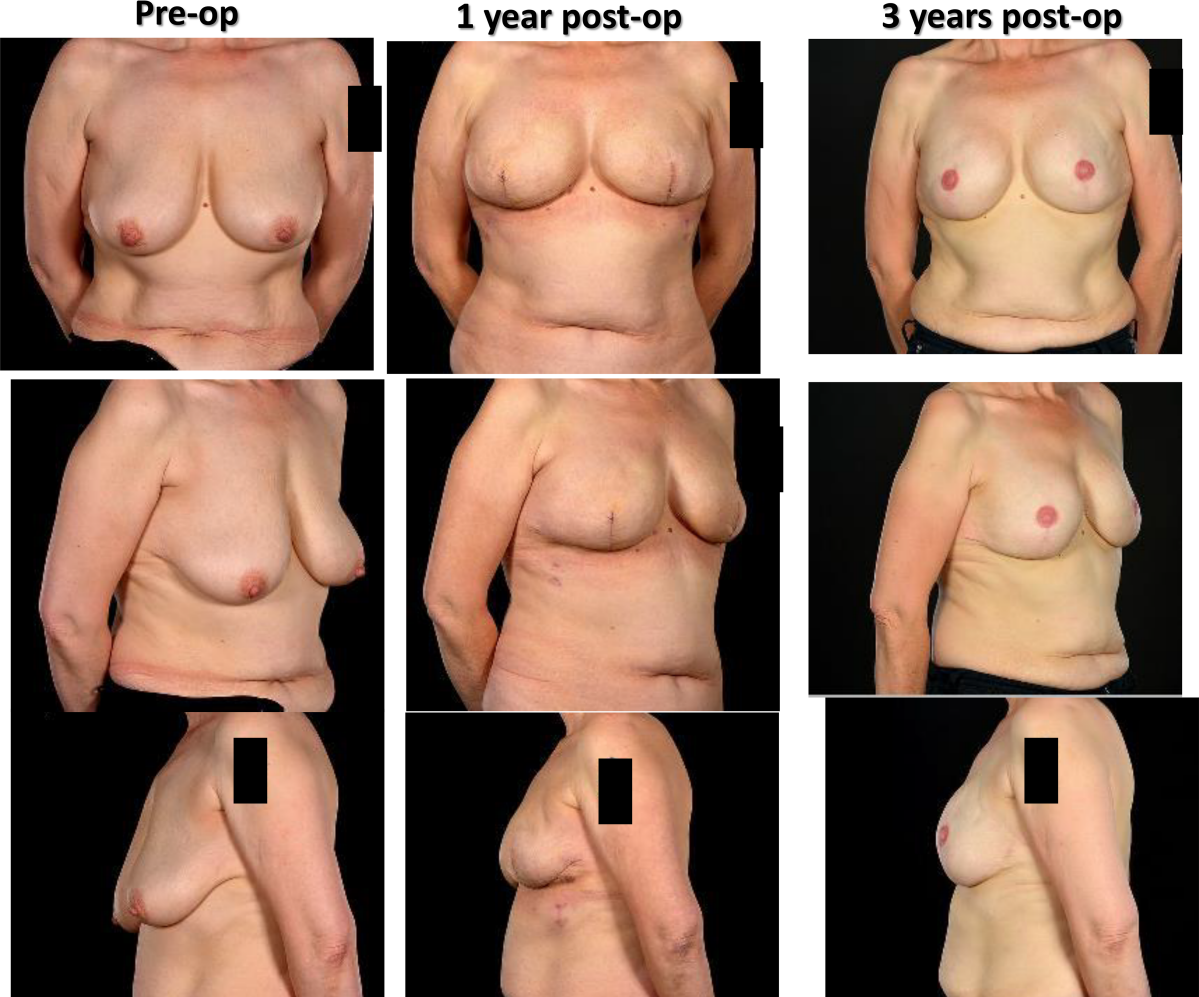
Skin reducing mastectomy, expandable implant and dermal sling reconstruction.

The technique for performing either skin- or nipple-sparing mastectomy should respect the oncological plane and ensure that dissection is confined to this plane between the anterior lamella of the superficial fascia and the subcutaneous tissue. It is crucial that subcutaneous blood vessels are preserved, and flaps are not too thin as this will compromise viability and lead to areas of flap necrosis. Adequate access is imperative to ensure that dissection continues to the extreme medial and superior limits of the breast; a separate axillary incision can be made if necessary to approach the ligament of Spence and axillary contents. Some patients require reduction of the breast skin envelope and this will usually be undertaken with a Wise pattern incision and occasionally an inferior dermal sling can be created to support the reconstruction (**Figure 12**).

## Autologous tissue versus implant-based reconstruction

Breast reconstruction can be performed using either prosthetic material or autogenous tissue whether this be IBR or DBR. It is recommended that patients meet with their breast surgeon early on to discuss options for reconstruction and be fully informed about these, especially with regards to various types of autologous reconstruction; patients may underestimate the complexity and risks of reconstructive surgery involving tissue transfer techniques.

Autologous tissue reconstruction is most commonly undertaken using flaps harvested from the lower abdomen or the upper back. Abdominal flaps comprise the transverse rectus abdominis myocutaneous (TRAM) flap and deep inferior epigastric artery perforator flap (DIEP) and the superficial inferior epigastric flap (SIEA). The upper back flaps based on the thoracodorsal vessels are the standard latissimus dorsi (LD) flap and the totally autologous LD flap. Other potential flap donor sites for breast reconstruction include the thighs (TUG/TMG, PAP, ALT flaps) buttocks (IGAP, SGAP flaps) posterior trunk (LAP flap) and iliac region (Ruben’s peri-iliac flap). Interestingly the use of the LD flap in conjunction with an implant (implant-assisted LD flap) for breast reconstruction has dramatically fallen in recent years with the advent of acellular dermal matrices that provide alternative coverage and support for an implant (86, 87). Similarly, a totally autologous LD flap (usually only possible in 15% of patients undergoing LD flaps) is now less frequently performed with emergence of pre-pectoral approaches to implant-based breast reconstruction that minimize animation and reduce post-operative pain. There has also been a reduction in the percentage of complex lower abdominal flaps for breast reconstruction.

The lower abdominal pannus provides generous tissue bulk for reconstruction purposes and therefore supplementation with an implant is unnecessary. These abdominal flap-based reconstructions have pedicle and free-flap variants and are more technically challenging with a greater risk of complications than implant only methods and may entail a microvascular anastomosis. Training in these autologous flap techniques is often protracted and involves specific microsurgical training (e.g. one year fellowship) and therefore these procedures are performed exclusively by plastic surgeons. The free-TRAM flap necessitates harvesting a variable amount of the ipsilateral rectus abdominis muscle (Nahabedian types 1-4) with consequent associated morbidity. The DIEP flap involves dissection and isolation of vascular perforators that pass from the inferior epigastric artery through to the rectus muscle fibres to supply the overlying skin and fat. The muscle therefore remains intact without compromise of abdominal wall integrity but retaining a large infra-umbilical pannus of skin and subcutaneous tissue with which to reconstruct the breast. The evolution of the free-TRAM into the DIEP flap has helped reduce donor site morbidity but there remains a finite risk of complete flap failure. This is generally between 3% and 5% and surgeon-specific rates should be made available to patients who may then chose not to embark on these higher risk reconstructive procedures. Rates of flap failure as low as 3% are achievable in units with high volume throughput and dedicated plastic surgeons with a predominant interest in breast reconstruction. Pre-operative CT angiography has helped predict the chance of success with a DIEP flap reconstruction by identifying candidate perforators and conversely indicating those patients who are technically unsuitable for this procedure. The biggest advantage of CT angiography in breast reconstruction has been to speed up the surgery (88) as it provides an intraoperative roadmap for perforator selection, location and dissection.

**Figure 13:**
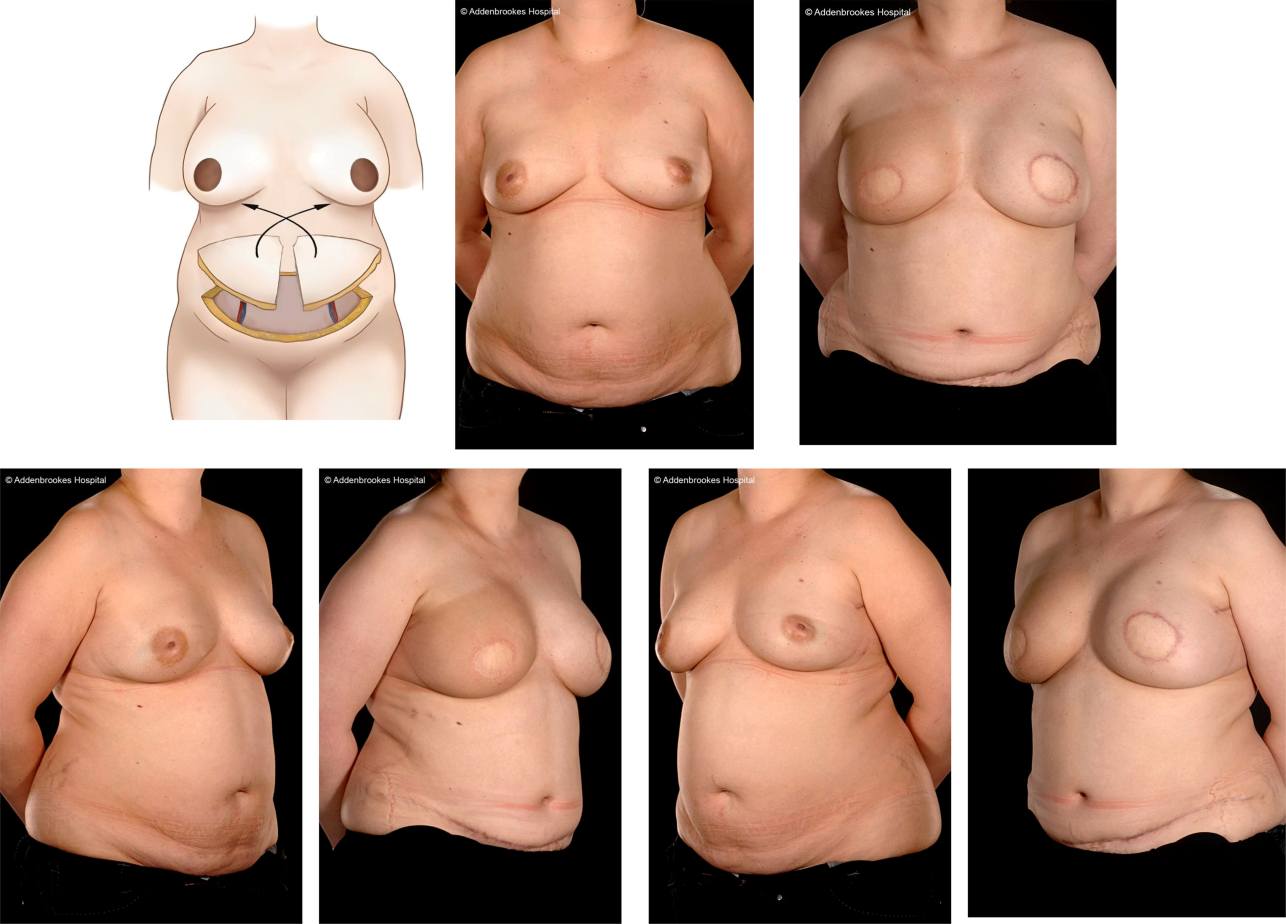
Bilateral DIEP flap immediate breast reconstruction following right therapeutic and left prophylactic skin sparing mastectomies. Illustration by Shiuan Shyu.

Implant-based procedures are the most commonly performed type of reconstruction worldwide and constitute about 70% of breast reconstructions in the UK (and a progressively increasing proportion of cases in the United States). This represents a simpler reconstructive option and has a more acceptable risk profile for many patients than autogenous forms of breast reconstruction. A particular advantage of implant-based IBR is a more rapid return to work and daily activities including familial and other commitments. Most women prefer to retain the same breast size but bilateral mastectomy and implant reconstruction provides the option for either downsizing or upsizing. This can also be possible with abdominal flap reconstruction depending on the relative sizes of the breasts and lower abdomen (**Figure 13**).

Implant-based reconstruction is technically easier and faster to perform but can be associated with an overall complication rate of 25%. A recent national audit was completed in the UK revealing a mean implant loss rate of 9% (89).

Key issues with implant-based reconstruction relate to type of implant (fixed volume or temporary expander or expandable implant), anatomical site (sub-pectoral or pre-pectoral), shape (anatomical or round) and implant size and to a lesser extent projection. More recently whether to use a smooth or textured surface prostheses has become important in the light of the causative association of implant surface texturing with ALCL.

Round implants provide uniform projection all around and especially in the upper pole. Rotation of such implants has minimal consequence in terms of cosmetic appearance. By contrast, anatomical/bi-dimensional implants are tear drop in shape and more closely mimic the natural anatomical shape of the breast. Their *in situ* rotation can have serious consequences for the cosmetic appearance by producing weird breast shapes. Fortunately, anatomical implants only come with textured surfaces which reduce malrotation, malposition and flicking over.

Fixed volume implants are exactly as their name implies and have an immutable size and volume compared with expander implants whose volume is adjustable *via* a port that is tunnelled subcutaneously. This can be minimally filled initially to relieve any pressure effects on the mastectomy flaps and wound during healing and can then be slowly expanded. Expanders can be temporary or permanent and ports can be removed separately if required (under local anaesthesia).

Fixed volume implants and tissue expanders can be placed in a sub-muscular location with complete muscle coverage (beneath a pocket formed from the pectoralis major and serratus anterior muscles). Today prostheses are generally placed in the subpectoral position with partial coverage in a dual plane setting with a combination of pectoralis major muscle and a piece of ADM that is sutured between the lateral border of pectoralis major and the chest wall (IMF) to complete the implant pocket. Increasingly, the implant is placed in a pre-pectoral (epipectoral) location without disruption of the pectoralis major muscle. In consequence there is no muscle animation, less post-operative pain and possibly evidence of less capsular contracture (90). A potential disadvantage of pre-pectoral implants is rippling which is more apparent in patients with a lower BMI and hence thinner flaps. The latter can be subsequently lipo-filled to improve overall contour of the flaps (increasingly oncoplastic surgeons are considering pre-pectoral reconstruction as a multiple stage procedure factoring in 2-3 rounds of postoperative fat grafting). Emerging data including a meta-analysis (90, 91) suggests there is no difference in patient reported outcome measures (Breast Q scores) between pre-pectoral and sub-pectoral implant reconstruction but significant differences in rates of capsular contracture, animation deformity and prosthesis failure favouring pre-pectoral placement of implants (92–94). In addition to issues such as pain and animation, dissection of a sub-pectoral pocket with elevation of the pectoralis major muscle in a dual plane approach with ADM can cause significant upper limb morbidity with associated arm weakness.

The current generation (5^th^) of implants are composed of material that is unlikely to cause an issue with longevity and necessitate routine replacement. Capsular contracture is an individual response of the host and exacerbated by exposure to irradiation which may require further surgery with capsulotomy and implant exchange. Breast implant associated anaplastic large cell lymphoma (BIA-ALCL) and breast implant-related illness are well publicized conditions which remain poorly understand but explanation of associated risks are part of the standard consent process. The risk of BIA-ALCL is commonly quoted as 1 in 28,000 but figures vary widely. This rare form of lymphoma arises in an effusion or scar capsule and patients are warned that any sudden breast swelling occurring more than 8-10 years from original surgery should be investigated to exclude BIA-ALCL. Fluid aspirated from around the implant can be tested for CD30 and the disease-specific marker ALK (anaplastic lymphoma kinase), reflecting a translocation in the tyrosine kinase receptor gene. Treatment in most cases is local and involves excision of the capsule and removal of the implant with later stages sometimes requiring systemic treatment with chemotherapy that is more effective for ALK positive cases (95).

Radiotherapy has a major impact on aesthetic outcomes following irradiation of skin and remaining breast parenchyma after OPBS and in the context of post-mastectomy radiotherapy (PMRT) with whole breast implant-based reconstruction. Although all cases of breast conservation will require radiotherapy, often a final decision on PMRT is made post-operatively when results of definitive histopathology are available. Nonetheless, PMRT may be anticipated, and this will be factored into the decision-making process in terms of surgical approach. Reports to-date suggest that ADM offers some protection from radiotherapy effects (96)and pre-pectoral reconstruction is not associated with significantly higher rates of capsular contracture following PMRT (94).

## Complications of oncoplastic surgery

The Dindo classification of surgical complications introduced in 2004 is widely employed in breast surgery and permits objective comparison of outcomes between different studies.

Complications relating to breast surgery patients are generally of mild to moderate severity and lie somewhere between grade 1 and grade 3b, grade 1 being a deviation from normal surgical course without the need for any type of intervention; grade 2 being a complication that requires pharmacological intervention or blood transfusion; grade 3a requiring surgical or radiological intervention without general anaesthetic (97). Nonetheless, these levels of complication can cause significant harm to a patient’s physical and mental well-being as well as trust in their surgeon. Neoadjuvant chemotherapy (NACT) patients often require some form of oncoplastic or reconstructive surgery and are more susceptible to surgical complications. The latter may lead to delays in radiotherapy (breast, chest wall, regional nodes) or systemic adjuvant treatments that are increasingly being used in patients with residual disease following NACT (e.g. capecitabine or CDK4/6 inhibitors). Major complications and problems with wound healing can impact significantly on long term breast cancer outcomes in terms of overall and disease-free survival and efforts should be made to minimize the incidence of any adverse surgical events. This includes appropriate selection of patients for more complex procedures, judicious use of surgical adjuvant materials and administration of antibiotics and thromboembolic prophylaxis.

Neoadjuvant chemotherapy approaches increase rates of breast conservation from down-sizing of tumors with rates of mastectomy reduced by 25% - 50% after induction chemotherapy (98, 99) Studies have shown a benefit to patients in terms of physical morbidity and psychological well-being from breast conservation surgery (BCS) compared with mastectomy which is also a more cost-effective treatment with significantly fewer surgical complications. Moreover, there are several other potential advantages for patients with larger lesions undergoing primary chemotherapy. They include *in vivo* determination of tumor sensitivity, eradication of micrometastatic disease with improved overall survival and downstaging of axillary nodes to de-escalate definitive surgery and minimize upper limb morbidity. Some patients have dramatic clinical response to standard pre-operative chemotherapy regimens and for patients with HER2 positive disease and triple negative cancers, complete pathological response rates can be as high as 70%. Those patients with a complete pathological response (pCR) in both the breast and axilla have improved longer term survival (100) but recent improvements in pCR have failed to translate into higher rates of breast conserving surgery (101). Furthermore, there is an increasing body of evidence that BCS and radiotherapy produces better overall survival outcomes compared with mastectomy (102). Nonetheless, a meta-analysis of clinical trials comparing neoadjuvant to adjuvant chemotherapy reported an increase in local recurrence for the breast conserving surgery group (99). The increase in local recurrence was greatest in the trials that included “no surgery” after NACT. When these 2 trials were excluded then there was only a 3.2% absolute increase in local recurrence in the NACT group at 10 years (RR 1.28, 95% CI: 1.06-1.55). During these trials there was no marking of the tumour site, so in patients with a complete imaging response who had residual disease, the surgeon performed an excision without guidance and is likely to have missed this residual disease in a significant number. It is for this reason that marking the tumour site is now standard practice and there is no reason to resect the original tumour footprint when there is radiological evidence of concentric shrinkage or no residual tumour is apparent. Techniques of OPBS may permit breast conserving surgery in large breasted patients with a ‘honeycomb’ pattern of shrinkage; there are many advantages of achieving, determining, and utilizing treatment response prior to surgical intervention and patients should be encouraged to undergo breast conserving surgery after neoadjuvant chemotherapy when indicated (103).

## Shared decision making

The clinical decision-making process for oncoplastic and reconstructive surgery is broadly based on a range of patient-related factors (including patient preference), surgeon-related factors (including preference and skills set) and healthcare resources (including availability of theatre/capacity).

Patients should be encouraged to engage in a shared decision-making process and will derive information from discussion with surgeons and breast care nurses often supplemented with online resources. They must be given sufficient time to absorb and process information before reaching a final decision on surgical management. Some studies have reported that only one-third of patients recalled being adequately informed about surgical options upon being questioned 3 months following surgery (104, 105). These have emphasized the importance of a balanced information giving process such that patients can make an informed decision regarding breast reconstruction without feeling overwhelmed. More than one-third (35%) of patients in one study who declined reconstruction stated that they considered the amount of information provided on surgical options was insufficient. In the study of Alderman et al, patients who were well informed of reconstructive options were four times more likely to undergo mastectomy (with IBR) than oncoplastic breast conserving surgery. Patients’ personal preference may be influenced by family, financial and social pressures depending on her particular circumstances and socio-economic background. Patients may have already formed a clear decision on what type of surgery they want but remain receptive to additional information; there is evidence that levels of patient satisfaction post-operatively are partly determined by the degree to which a surgeon influences a patient’s decision. Surgeons should strive to understand why patients chose a particular option and understand that most patients are motivated by body image issues such as “seeking to maintain a balanced appearance” (45).

More objective patient-related factors that influence suitability for an oncoplastic or reconstructive procedure include tumour: breast ratio, breast size and shape (degree of ptosis), body habitus, BMI, co-morbidities, anticipated adjuvant treatments, smoking habits, anatomical factors determining available tissue volume for breast reconstruction/replacement and previous radiotherapy.

Patient factors and co-morbidities that influence healing, recovery, and tolerance of surgical/adjuvant treatments (chemotherapy and radiotherapy) include factors such as: smoking, diabetes, high BMI, vasculopathy, connective tissue diseases. Other factors to consider are patient expectations and understanding of their disease and treatment together with the need for any psychological evaluation and support.

Breast cancer treatment involves a variety of modalities with possible side effects, which can be difficult for patients to understand and accept. One patient compared her journey through chemotherapy, surgery, and radiotherapy to being "poisoned, slashed, and burnt." She revealed that she was unprepared for the whirlwind experience and its considerable impact. From a holistic perspective, breast cancer treatment affects patients' families, relationships, work, sexuality, confidence, independence, and mortality perception. With deadlines looming, patients must overcome obstacles and make decisions, even though they recognize that their choices may affect their survival. It is not unreasonable for patients to question the consequences of failure.

As we have all seen once patients cross that initial phase of treatment and suddenly their chemotherapy and radiotherapy has finished, they are left with the one visible reminder of their journey; the breast. It can be a conflicting entity, a symbol of their sexuality, motherhood, cancer, and mortality. Many studies have confirmed that better aesthetic outcomes improve quality-of-life. The aforementioned catalogue of oncoplastic and reconstructive procedures provide breast cancer patients with a variety of options that can maintain or improve overall well-being and quality-of-life.

Future directions of oncoplastic and reconstructive breast cancer surgery includes the sharing of information on a national and international level to identify trends and learn from good practice. BCCT.core software can help breast surgeons evaluate cosmetic outcomes of OPBS more objectively and 3D imaging for reconstruction can help with the consent and planning process (106). As we continue to perfect aesthetic outcomes the next frontier in OPBS may be sensation preservation (107).

Other patient-related factors that impact wound healing, post-operative recovery and how well patients tolerate adjuvant treatments include diabetes (types I and II), vasculopathy and connective tissue disorders. These latter conditions can affect viability of mastectomy flaps and transposed glandular tissue of the breast and some connective tissue diseases are a relative contraindication to radiotherapy.

## Concluding comments 

The diagnostic and treatment pathway for breast cancer patients is a difficult journey psychologically and it is important to gauge patients’ understanding of their disease and in particular expectations from breast reconstructive surgery. Patients should not undergo simultaneous contralateral prophylactic mastectomy without formal psychological assessment. There is a relatively restricted timeline once a tissue diagnosis of breast cancer has been made and this may prove challenging and overwhelming for some patients who need more time to adjust to the diagnosis and accept a management plan that is likely to involve multi-modality treatment. Patients must cope with the mutilating effects of surgery and adverse side-effects of radiotherapy and systemic treatments (that usually include hair loss – a major concern and frequent source of alarm for many younger patients). A breast cancer diagnosis has wider implications for a patient’s family and relationships be this work-related or more intimate. Apart from negatively affecting body image, self-confidence and sense of femininity, breast cancer is a potentially life-threatening disease and can abruptly remind a patient of their own mortality. Survivorship has come to the forefront in recent years with more attention to quality-of-life and minimising the sequelae of breast cancer treatments – especially surgery and radiotherapy. Patients should have not only an acceptable cosmetic result, but also optimal functional outcomes without chronic symptoms of niggling discomfort or more overt pain symptoms. Nonetheless, the majority of women are successfully treated and will live for many years following breast cancer treatment.

## Author contributions

PW and JRB wrote the article. JRB edited article. CMM edited second part of the article, contributed photography and diagrams. All authors contributed to the article and approved the submitted version.
